# The kinematics of handwriting movements as expression of cognitive and sensorimotor impairments in people with multiple sclerosis

**DOI:** 10.1038/s41598-017-18066-7

**Published:** 2017-12-18

**Authors:** Ambra Bisio, Ludovico Pedullà, Laura Bonzano, Andrea Tacchino, Giampaolo Brichetto, Marco Bove

**Affiliations:** 1 Department of Experimental Medicine, Section of Human Physiology, University of Genoa, Genoa, Italy; 2 Department of Neuroscience, Rehabilitation, Ophthalmology, Genetics, Maternal and Child Health, University of Genoa, Genoa, Italy; 3 Scientific Research Area, Italian MS Foundation, Genoa, Italy; 4 Italian MS Society Rehabilitation Centre, Genoa, Italy

## Abstract

Handwriting is an important activity of daily living, which requires sensorimotor and cognitive skills that could deteriorate in presence of neurological diseases. Handwriting impairments are common in people with multiple sclerosis (PwMS). Aims of the present study were to characterize handwriting movement features of PwMS in comparison with those of healthy adults, and to evaluate the relationship between kinematic parameters of handwriting movements and the results of the assessment of cognitive and motor domains. A new handwriting evaluation methodology was applied to quantify handwriting features of 19 PwMS and 22 age-matched healthy controls who were required to write a sentence on a digitizing tablet. Kinematic parameters of the sentence and of the strokes were used to evaluate handwriting performance. PwMS showed an altered handwriting kinematics with respect to healthy controls: higher movement duration, fragmented velocity profile and higher jerk. Furthermore, motor abilities and cognitive status of PwMS were related to handwriting parameters in accordance with the evidence that MS is a multifactorial disease affecting different domains. These results suggested that the proposed methodology might be a valuable tool to quantitatively assess handwriting impairments and the efficacy of handwriting treatments in PwMS.

## Introduction

One of the common clinical features of people with multiple sclerosis (PwMS) is impaired hand function caused by deterioration of hand dexterity^1^, and thus problems in actions involving a fine control of fingers movements. Objects manipulation^2^ and finger-tapping movements^3^ are two examples where PwMS showed motor impairments as a result of their deficiencies in sensitivity, muscle strength and coordination. Also, handwriting deficits are frequently encountered in MS^4–7^. Handwriting is an important and high-value activity, which requires complex sensorimotor, perceptual and cognitive skills^8,9^. If one of these abilities declines, as frequently occurs in neurological diseases, such as Parkinson’s disease and MS, handwriting could deteriorate causing a sense of frustration and the inability to recognize themselves in the new calligraphy. A number of researches investigated handwriting skills in Parkinson’s disease, showing significant impairments in maintaining a given size and speed that result in micrographia and bradykinesia, probably related to impairment in force control^10–16^. Differently, the scientific literature dealing with the behavioral evaluation of handwriting in PwMS is limited to few studies.

The first work addressing this issue dates from the beginning of the 1990s, when Wellingham-Jones^4^ compared a group of PwMS with a group of control subjects using the Roman-Steaempfli Psychogram, a graphological charting system. As hypothesized by the author, the results showed that patients’ fluency, rhythm and control in script were lower than in healthy participants, and there was an increase of the irregularity, narrowness, slowness, tremor and distortion.

About ten years later Schenk and coworkers^5^, using a digitizing tablet, showed that the handwriting kinematic features of PwMS were altered; a reduced writing speed corresponded to an increased stroke duration and an irregular and multi-peaked velocity profile.

Furthermore, Longstaff and Heath compared the kinematics of a group of PwMS while drawing spirals with that of a group of healthy volunteers and found in PwMS a significant increase in trajectory variability, and a tendency to draw slower and with less pen pressure than controls; these results highlighted fine motor control degradation^7^.

These studies pointed out the relationship between handwriting and sensorimotor impairments, disregarding how cognitive deficits, commonly present in PwMS^17^, might influence the writing task. For this reason, the aims of the present study were to characterize handwriting movement features of PwMS in comparison with those of healthy adults, and to evaluate the relationship between kinematic parameters of handwriting movement and the results of the assessment of the cognitive and the motor domains. To do that a group of PwMS and a group of healthy age-matched controls (HC) were recruited and were asked to write three times at spontaneous speed, over a digitizing tablet, the Italian sentence “Il sole scalda” (i.e., “The sun warms”). This method was chosen among others, as for instance “ll” writing^12,13,18^ and spirals drawing^7,15,19^, in order to evaluate the handwriting performance during an ecological writing task, when the subject writes a meaningful sentence rather than drawing meaningless form or pseudo-words. This might also be an advantage when looking for the relationship between cognitive status and handwriting performance.

The handwriting performance was evaluated on the sentence as a whole, on words and spacing between words. The quality and the level of automation of handwriting was checked by means of the single stroke analysis^20,21^. The kinematic parameters were compared between PwMS and HC groups. Furthermore, the correlations among kinematic parameters and the score of the clinical evaluations were assessed in PwMS.

## Results

### Comparison between PwMS and HC

#### Analysis of the sentence

The handwriting movement kinematic parameters and the coefficient of variation (CV) of the sentence were evaluated by means of MANOVAs. The results on handwriting parameters showed a significant difference between the two groups (*F(7*,*33)* = 2.*58*, *p* < *0*.*05*, *ƞ* ^2^ = *0*.*75*). In detail, group differences were found in sentence duration (*F(1*,*39)* = *13*.*83*, *p* = *0*.*001*, *ƞ* ^2^ = *0*.*95*), words duration (*F(1*,*39)* = *9*.*44*, *p* = *0*.*004*, *ƞ* ^2^ = *0*.*85*) and spacing between words duration (*F(1*,*39)* = *8*.*78*, *p* = *0*.*005*, *ƞ* ^2^ = *0*.*8*2), with significantly longer time spent by PwMS with respect to HC. No significant differences were found between the two groups in sentence length and height, in words length and in length of the spacing between words. Concerning movement variability, the result of the MANOVA did not show significant differences between the two groups.

#### Analysis of the stroke

The MANOVAs on stroke kinematic parameters showed a significant effect of the factor group (*F(5*,*35)* = *5*.*59*, *p* < *0*.*001*, *ƞ* ^2^ = *0*.*98*). The follow-up tests indicated that the group significantly affected each dependent variable. In detail, significantly higher stroke duration (*F(1*,*39)* = *15*.*91*, *p* < *0*.*001*, *ƞ* ^2^ = *0*.*97*), stroke size (*F(1*,*39)* = *7*.*3*2, *p* = *0*.*01*, *ƞ* ^2^ = *0*.*75*), normalized jerk (*F(1*,*39)* = *17*.*71*, *p* < *0*.*001*, *ƞ* ^*2*^ = *0*.*98*) and number of inversion in the velocity profile (NIV) (*F(1*,*39)* = *12*.*23*, *p* = *0*.*001*, *ƞ* ^*2*^ = *0*.*93*) were observed in PwMS than in HC. Furthermore, the number of stroke (NoS) increased in PwMS with respect to HC (*F(1*,*39)* = *6*.*11*, *p* = *0*.*018*, *ƞ* ^*2*^ = *0*.*67*), despite p-value was slightly higher than the Bonferroni’s corrected p-value (*p* = *0*.*01*).

**Figure 1:**
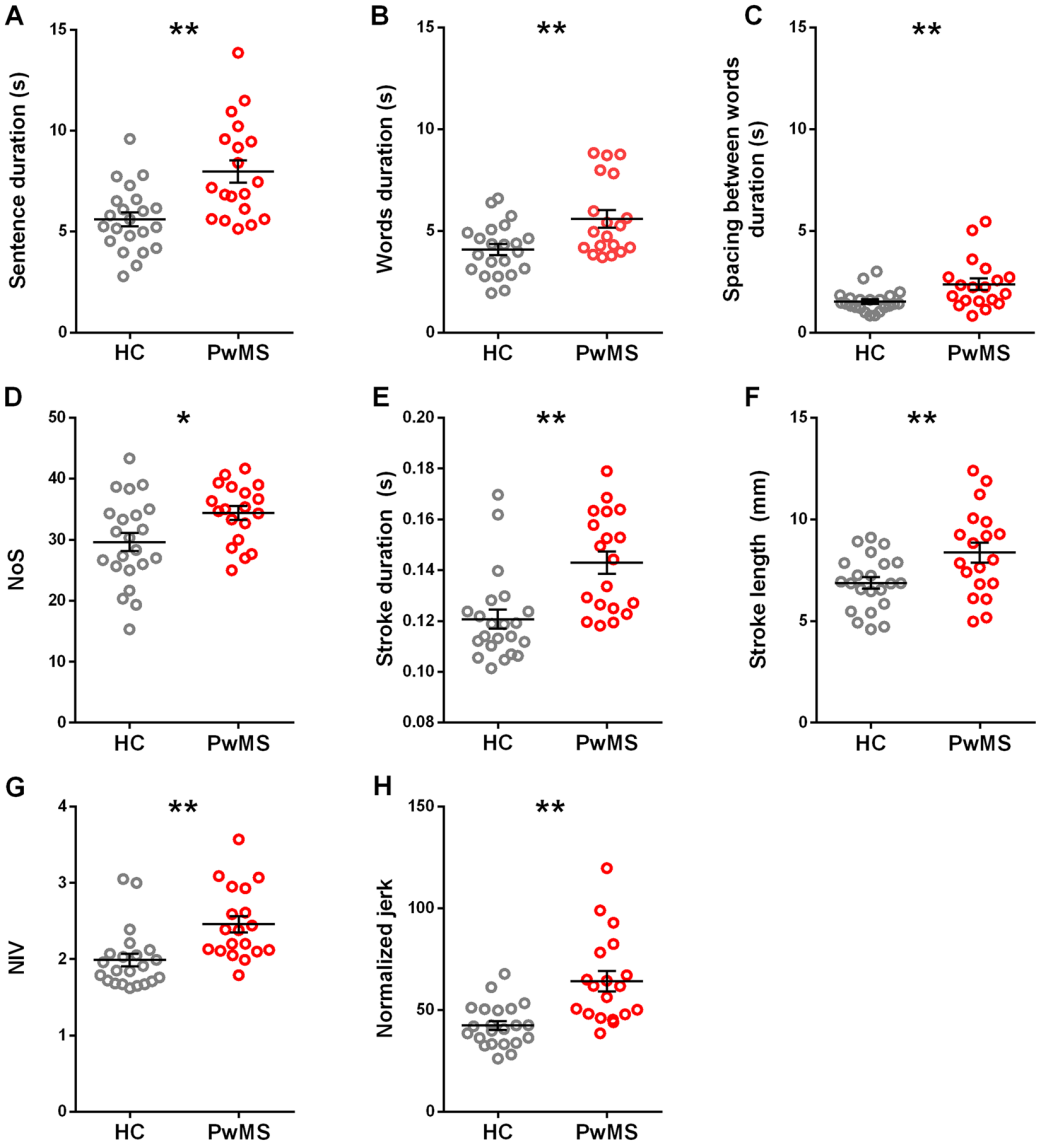
Handwriting kinematic parameters of healthy controls (HC – grey circles) and people with multiple sclerosis (PwMS, red circles). The first line shows the sentence parameters: duration of the sentence (**A**), words (**B**), and spacing between words (**C**). The second and third lines show stroke parameters: number of strokes (NoS - **D**), stroke duration (**E**) and length (**F**), number of inversion in the velocity profile (NIV - **G**), normalized jerk (**H**). Each circle represents the average value for a single subject. The horizontal line indicates the group average, and the error bars show the standard error value. * and **indicate p < 0.05 and p < 0.01, respectively.

Both the sentence and the stroke parameters of PwMS and HC are reported in Table 1. The parameters showing significant differences between groups are also represented in Fig. 1.

**Table 1:** Kinematic measurements of the sentence and of the strokes.

	**HC**	**PwMS**	**Statistics**
**Sentence parameters**			
Sentence duration (s)	5.61 ± 0.34	7.98 ± 0.58	*p* < *0*.*001*
Sentence length (mm)	89.16 ± 4.99	92.77 ± 5.60	*n*.*s*
Sentence height (mm)	11.14 ± 0.78	12.68 ± 1.23	*n*.*s*
Words duration (s)	4.08 ± 0.27	5.59 ± 0.44	*p* = *0*.*004*
Words length (mm)	66.31 ± 4.04	70.56 ± 4.18	*n*.*s*
Spacing between words duration (s)	1.53 ± 0.11	2.38 ± 0.29	*p* = *0*.*005*
Spacing between words length (mm)	22.86 ± 1.36	22.34 ± 2.08	*n*.*s*
CV of the sentence duration	0.11 ± 0.05	0.19 ± 0.05	*n*.*s*
CV of the sentence length	0.04 ± 0.006	0.07 ± 0.01	*n*.*s*
CV of the sentence height	0.11 ± 0.01	0.14 ± 0.02	*n*.*s*
CV of the words duration	0.12 ± 0.02	0.15 ± 0.02	*n*.*s*
CV of the words length	0.06 ± 0.01	0.09 ± 0.01	*n*.*s*
CV of spacing between words duration	0.18 ± 0.05	0.28 ± 0.08	*n*.*s*
CV of the spacing between words length	0.09 ± 0.01	0.15 ± 0.02	*n*.*s*
**Stroke parameters**			
Number of strokes - NoS	29.63 ± 1.51	34.40 ± 1.11	*p* = *0*.*02*
Stroke duration (s)	0.12 ± 0.003	0.14 ± 0.004	*p* < *0*.*001*
Stroke size (mm)	6.88 ± 0.28	8.37 ± 0.49	*p* = *0*.*01*
Normalized jerk	42.36 ± 2.23	64.18 ± 4.95	*p* < *0*.*001*
Number of inversions in velocity - NIV	1.99 ± 0.08	2.46 ± 0.11	*p* = *0*.*001*
CV of NoS	0.7 ± 0.01	0.7 ± 0.01	*n*.*s*
CV of the stroke duration	0.39 ± 0.01	0.40 ± 0.02	*n*.*s*
CV of the stroke size	0.68 ± 0.02	0.67 ± 0.02	*n*.*s*
CV of the normalized jerk	0.11 ± 0.01	0.12 ± 0.02	*n*.*s*
CV of NIV	0.06 ± 0.01	0.07 ± 0.01	*n*.*s*

Data are reported as mean ± standard error. No significant differences between healthy controls (HC) and people with multiple sclerosis (PwMS) are marked as “n.s.”.

### Relationship between clinical scores and kinematic parameters

Table 2 shows the correlations between clinical scores and kinematic parameters that resulted to be significant. Eight multiple linear regression (MLR) models were apply to correlate the kinematic parameters that resulted to be significantly altered in PwMS with the results of the clinical evaluation.

**Table 2:** Results from the correlation analysis between kinematic parameters and clinical scales evaluations (only significant correlations are reported).

	**Clinical evaluation parameter**	**β**	**partial R** ^**2**^	**partial p**
**Sentence parameters**				
Sentence duration vs.	MLR model: R = 0.85; R^2^ adjusted = 0.62; p = 0.003			
	NHPT	*0*.*84*	*0*.*38*	*0*.*001*
	Grip strength	−*0*.*72*	*0*.*76*	*0*.*03*
	Pinch tri-pod strength	*0*.*75*	*0*.*79*	*0*.*03*
	MFIS tot	−*0*.*37*	*0*.*12*	*0*.*03*
	Non-parametric (Spearman’s) correlation			
	VAS for weakness		*0*.*55*	*0*.*02*
Words duration vs.	MLR model: R = 0.84; R^2^ adjusted = 0.58; p = 0.004			
	NHPT	*0*.*57*	*0*.*38*	*0*.*01*
	SDMT	−*0*.*49*	*0*.*32*	*0*.*02*
	Grip strength	−*0*.*77*	*0*.*76*	*0*.*03*
	Pinch tri-pod strength	*0*.*76*	*0*.*79*	*0*.*03*
	MFIS tot	−*0*.*42*	*0*.*12*	*0*.*02*
Spacing between words duration vs.	MLR model: R = 0.72; R^2^ adjusted = 0.34; p = 0.04			
	NHPT	*0*.*82*	*0*.*38*	*0*.*005*
	Non-parametric (Spearman) correlation			
	VAS for weakness		*0*.*58*	*0*.*01*
**Stroke parameters**				
Stroke durations vs.	MLR model: R = 0.81; R^2^ adjusted = 0.53; p = 0.008			
	NHPT	*0*.*81*	*0*.*38*	*0*.*001*
	Pinch tri-pod strength	*1*.*14*	*0*.*79*	*0*.*006*
	MFIS tot	−*0*.*41*	*0*.*12*	*0*.*03*
NIV vs.	MLR model: R = 0.85; R^2^ adjusted = 0.72; p = 0.003			
	NHPT	*0*.*78*	*0*.*38*	*0*.*02*
	Grip strength	−*1*.*04*	*0*.*76*	*0*.*004*
	Pinch tri-pod strength	*1*.*22*	*0*.*79*	*0*.*002*
	MFIS tot	−*0*.*42*	*0*.*12*	*0*.*02*
Normalized jerk vs.	MLR model: R = 0.86; R^2^ adjusted = 0.74; p = 0.002			
	NHPT	*0*.*87*	*0*.*38*	<*0*.*001*
	Grip strength	−*0*.*83*	*0*.*76*	*0*.*01*
	Pinch tri-pod strength	*1*.*10*	*0*.*79*	*0*.*003*
	MFIS tot	−*0*.*42*	*0*.*12*	*0*.*02*

In particular, the dependent variables sentence, words, spacing between words durations, NoS, stroke duration and length, normalized jerk and NIV were correlated with NHPT, MFIS, SDMT, grip and pinch forces. Spearman’s correlation analyses were applied to evaluate whether the same dependent variables were correlated with VAS for weakness, sensory impairment and coordination problems, and with AbilHand score. Non-significant correlations (p > 0.05) were not described in the text and not included in Table 2.

#### Correlation between clinical scores and sentence parameters

The multiple linear regression (MLR) models showed that the sentence duration negatively correlated with the MFIS score (i.e., the higher the fatigue perceived in the month preceding the evaluation, the lower the duration of the sentence) and with the grip strength (i.e., the lower the strength of the grip, the longer the duration of the sentence), and positively correlated with the tri-pod pinch strength (i.e., the higher the tri-pod pinch strength, the longer the sentence duration) and the duration of NHPT (i.e., longer time to complete the NHPT corresponded to longer handwriting movement duration). Furthermore, a significant negative correlation was found with the SDMT scores, meaning that higher performance to this test corresponded to shorter sentence duration. The results of the Spearman’s correlations showed a significant positive relationship between sentence duration and VAS for weakness score, suggesting that PwMS reporting higher weakness were slower with respect to those who reported a lower VAS score. The time spent to write the three words negatively correlated with the MFIS score, the grip strength and the SDMT score, whilst it positively correlated with the tri-pod pinch strength and the duration of the NHPT. The duration of the spacing between words positively correlated with the VAS for weakness.

#### Correlation between clinical scores and stroke parameters

The result of the MLR model with stroke duration as dependent variable showed positive correlations with the duration of the NHPT and the pinch tri-pod strength, and a negative correlation with the MFIS score.

The NIV and the normalized jerk were significantly predicted by duration of the NHPT (positive correlation), the grip strength (negative correlation), the pinch tri-pod strength (positive correlation) and the MFIS score (negative correlation).

## Discussion

This study showed that handwriting movements of PwMS and HC significantly differed in the time spent to write the sentence, which was due to an increased duration of the words and of the spacing between words. In agreement, the duration of strokes was significantly higher in PwMS than in HC. Furthermore, strokes in PwMS were characterized by a significant increase in vertical stroke size, normalized jerk and number of inversion of the velocity profile with respect to HC. No differences were found in the coefficient of variability associated to all the analyzed kinematic variables.

Concerning the temporal component of the handwriting task, the scientific literature documented the increase in the time spent to perform motor tasks involving the lower as well as the upper limbs in PwMS^22,23^. Indeed, although walking impairment is a clinical hallmark of MS^24^, dysfunctions of the upper limb are also common^1,25^. In a descriptive cross-sectional study, where the time to complete the NHPT was used as measure of manual dexterity, the 76% of the patients over a wide range of EDSS reported disabilities^26^. In agreement with this work, Holper and colleagues showed that more than the 50% of 205 PwMS reported impairment or restriction to upper limb functions^27^. Altered performances have been reported in movements involving the proximal part of the arms^28^, in gross manual dexterity exercises^29^ and complex coordination tasks involving fine motor abilities^30^. Concerning this last point, Bonzano and colleagues quantified fingers motor impairments in PwMS even with very low disability, and found decreased spontaneous movement rate and altered performance in the bimanual coordination task^3^, which was previously found to be related to the loss of microstructural integrity of the corpus callosum^31–33^.

Handwriting, a kind of movement that requires fine motor control abilities, deteriorates in MS, and modifications with respect to healthy adults always include the increase in movement duration^5,34^, in agreement with the present results. The increase in sentence duration might be ascribed to impairments in two different domains, the cognitive and the motor domains, as also suggested by the correlations found with the clinical scores. The results of the multiple linear regression model showed that an increase in sentence duration corresponded to a longer time to complete the NHPT. When looking at the two components of the sentence, the relationship was found to be significant both with the duration of the words and with the time spent passing from one word to the following one. This result is confirmed by the analysis on the least sub-unit of the handwriting, namely the stroke: the longer the stroke duration, the longer the time to complete the NHPT. A recent review suggested the NHPT as a gold standard metric for measuring the manual dexterity in PwMS^30^ and the present results suggested that it is also related to the handwriting performance. Furthermore, the NHPT positively correlated also with the NIV and the normalized jerk, two kinematic parameters that were significantly greater in PwMS than in HC. Under spontaneous conditions, central processing of writing movements employs mainly feed-forward mechanisms, and sensory feedback is used to monitor the motion range^35,36^. Skilled writers produced strokes that resemble ballistic movements, characterized by a single-peaked, bell-shaped velocity profile (NIV = 1)^18,21^. However, sensorimotor control deficits, already described in PwMS^23,37^, may alter either feed-forward or feedback control mechanisms, or both at the same time. As a result, modifications to the handwriting kinematics could occur: namely, an increased number of inversions in the velocity profile, a reduction in movement’s smoothness (i.e., increased jerk value) and an augmented handwriting duration, features that characterized PwMS and distinguished them from HC in the present study. Therefore, we could suggest that some of the differences observed in handwriting performance between the two groups are motivated by deficient motor control strategies in PwMS when executing this semi-automated movement. In particular, these results might suggest the incapacity to maintain the initial motor plan throughout its course, hence requiring several online adjustments to the initial planned trajectory, as also documented in other neurological conditions when performing upper limb movements^38–42^. At the same time, the frequent corrections of movement direction might be related to a dysfunction in sensory input integration^43^ and/or to the attempt to compensate for poor proprioceptive control of upper limb using visual feedback^44,45^.

Nevertheless, it should be considered that handwriting is not merely a manual dexterity task relying only on motor strategies. Looking at the writing component, the duration of the words increased with the decrease in the SDMT score. The SDMT investigates the information processing speed, which is shown to be not only the cognitive domain most widely affected by MS but also the first cognitive deficit to emerge^46^. Recent studies suggested that the SDMT represents by itself a useful screening tool to measure cognitive impairments in MS since it showed high sensitivity and specificity in predicting the outcome of a complete neuropsychological test battery^47,48^. The present relationship between words duration and the SDMT score confirms that writing is not just a motor act but is strictly linked to the cognitive domain and showed, for the first time, that the cognitive status might impact PwMS handwriting abilities. It is worth noting that the task proposed in the present work was considered very easy by the participants since they had to write a previously memorized simple and short sentence, and none of them failed to report it. Nonetheless, the performance deteriorated as a function of cognitive impairment, indicating that a simple handwriting task could be affected by cognitive deficits, even subtle or minimal as in the case of the most of the PwMS enrolled in this study (according to the Italian normative values provided by Amato and colleagues^49^). This correlation was specific for the words, i.e., the written and meaningful component of the sentence, and not for the spacing between words, which was mainly related to the motor domain, as suggested by the significant positive correlation showing that the higher the perceived weakness, the longer the time spent between one word and the following one. Furthermore, the lack of significant relationships between the SDMT score and any of the analyzed stroke parameters suggested that, although stroke analysis is a commonly used technique to evaluate handwriting impairments^5,50–52^, it does not allow to appreciate handwriting in its entirety. Combining the sentence and the stroke analysis could provide a more complete description of handwriting^42^.

The duration of the sentence as a whole, and in particular of the words, was related to grip and pinch strength values. The grip strength is often used as an indicator of overall body muscular strength since it is easy to measure and correlates with the total muscle strength^53^. Here, increasing grip strength corresponded to fast writing movements, confirming previous evidences of the influence of muscle strength on movement speed both in healthy^54^ and in patients^55^ populations.

Conversely, a positive correlation was found between pinch strength and the duration of the sentence, words and strokes. At a first glance, these correlations seem difficult to explain, since several studies in healthy people, as well as in PwMS, suggested a positive correlation between pinch and grip strength^56–60^. One might speculate about the existence of a tradeoff mechanism between the force the participant can exert on the pen in order to stabilize it and the writing velocity; specifically, in order to produce a legible trace the participants who were able to produce a high force value might have devoted their effort to stabilize the pen at the cost of reduced writing speed, high fragmentation in the velocity profile (positive correlation between pinch strength and NIV), and decreased movement smoothness (positive correlation between pinch strength and normalized jerk). However, only few studies in children with handwriting difficulties^61,62^ and in writer’s cramp patients population^50,63^ analyzed the relationship between writing kinematics and the pinch strength. Future studies in PwMS will have to shed light on this aspect of handwriting.

At last, the fatigue perceived by PwMS influenced handwriting kinematics. In particular, sentence, words and stroke durations negatively correlated with the MFIS score, meaning that greater writing movement velocity was related to higher perceived fatigability. Furthermore, looking at the number of inversions and the normalized jerk, movements of PwMS with higher MFIS score were smoother (i.e., low NIV value) and less segmented (i.e., low normalized jerk value) than those of PwMS reporting lower subjective fatigue levels. These results, which might seem to be a paradox, are in agreement with a previous study of our group in PwMS investigating the relationship between fatigue and motor performance during the execution of a finger motor task paced with a metronome^64^. In this work, higher temporal accuracy (i.e., better motor performance) was associated with higher level of subjective fatigue and, in turn, PwMS that reported higher fatigue showed increased activity in the prefrontal and cerebellar areas. One might speculate that the fatigue-motor performance paradox here observed during handwriting might be related to the alteration of the neural circuitry involved in the perception of fatigue and in the effort-rewards system^65^.

## Conclusion

In a review dealing with rehabilitative procedures in MS it has been raised the necessity to develop new objective outcome measures able to evaluate the efficacy of rehabilitative interventions in a more accurate and personalized way^66^. In the present study, we applied a new handwriting evaluation methodology to quantify handwriting impairments in PwMS. An increase in movement duration and a deterioration of other kinematic parameters (NoS, stroke size, NIV, normalized jerk) were observed in PwMS with respect to healthy controls. Furthermore, for the first time, the results showed the influence of motor and cognitive status on handwriting skills. These findings might be very useful when planning rehabilitative task-oriented interventions focused on handwriting abilities; indeed, these results could suggest to evaluate both the motor and the cognitive status of PwMS in order to tailor the intervention on the most affected domain. Furthermore, this methodology might be considered as a valuable tool to quantitatively describe the efficacy of treatments targeting handwriting movement in PwMS.

## Methods

### Participants

Twenty-two PwMS reporting handwriting impairments during a preliminary brief interview were enrolled. However, since three of them did not complete all the evaluation procedure, the results of 19 PwMS (12 females, age range 26–60; mean age ± SD = 46 ± 11 years; mean ± SD disease duration 15.2 ± 9.9 years; 14 subjects had relapsing-remitting –RR – course and 5 subjects secondary progressive – SP - course) are presented. Further details concerning the occupation, MS phenotype, EDSS, disease duration, assumption of disease-modifying drugs, and affected limb are provided in Table 3.

**Table 3:** Demographic and clinical characteristics of the patients included in the study.

ID	Age (years)	Gender	Occupation^a^	MS phenotype	EDSS	Disease duration (years)^b^	Disease-modifying drug	Affected upper limb
1	53	F	Domestic worker	RR	1.5	5	—	Left
2	32	F	Salesgirl	RR	4	8	Fingolimod	Left
3	61	M	Pensioner	SP	6	8	Azatioprina	Left
4	62	M	Pensioner	SP	6	41	Methotrexate	Bilateral
5	44	F	Physical therapist	RR	2	19	Fingolimod	Right
6	43	F	Unemployed	RR	6	23	Interferon	Right
7	57	M	Pensioner	SP	6	19	Interferon	Right
8	53	M	Gardner	RR	5	13	—	Right
9	31	F	Employee	RR	5	8	Natalizumab	Bilateral
10	47	F	Storekeeper	RR	2	10	Interferon	Bilateral
11	62	M	Pensioner	RR	5	6	Fingolimod	Left
12	55	F	Secretary	RR	4	18	Interferon	Bilateral
13	36	F	Employee	RR	2	9	—	Left
14	43	F	Barmaid	SP	2	25	Fingolimod	Bilateral
15	40	F	Employee	RR	2	10	Fingolimod	Right
16	52	M	Pensioner	SP	6	32	—	Left
17	27	M	Bricklayer	RR	2	5	—	Bilateral
18	46	F	Housewife	RR	4	22	Fingolimod	Bilateral
19	29	F	Employee	RR	1	8	Fingolimod	Bilateral

RR = relapsing–remitting; SP = secondary progressive; ^a^in the last 10 years; ^b^from diagnose.

Furthermore, 22 healthy controls (HC; 16 females, age range 27–62, mean age ± SD = 39 ± 13 years) participated in this study. In the last 10 years their occupations were: employee (n = 5), salesperson (n = 3), beautician (n = 1), call-center operator (n = 1), secretary (n = 2), housewife (n = 2), domestic worker (n = 1), medical doctor (n = 1), warehouse worker (n = 1), pensioner (n = 2) and unemployed (n = 3).

PwMS were enrolled by the Rehabilitation Centre-Italian Multiple Sclerosis Society, Genoa. Inclusion criteria were the following: impairment in handwriting; both sexes; age major than 18 years; patients with definite MS according to the McDonald criteria^67^ in a stable phase of the disease; mild and moderate muscle strength deficit in the upper limb as assessed by the Medical Research Council scale^68^ (muscle strength with grade 4 in all muscle groups or grade 3 in no more than 2 joints). Exclusion Criteria were: a relapse in the last three months; Mini Mental State Examination^69^ minor than 26; Modified Ashworth scale to evaluate muscle tone of the upper limb^70^ major than 3 in at least 2 muscle groups; inability to perform simple handwriting movements. To avoid biases due to occupation, an additional exclusion criterion was a job requiring intensive daily handwriting.

All subjects recruited for this study were naïve to the specific purpose of the research and right-handed according to the Edinburgh Handedness Inventory^71^.

In addition, all the included patients were evaluated with the following clinical scales: Expanded Disability Status Scale (EDSS)^72^; manual muscle strength test with dynamometer to assess tri-pod pinch strength and grip strength; goniometer to assess degrees of active range of movement (ROM) for shoulder, elbow, wrist and fingers against gravity; Nine Hole Peg Test (NHPT)^73^ for hand dexterity; AbilHand scale^74^; Visual Analogue Scale (VAS) for self-perception of upper limb disability (weakness, sensory impairment and coordination problems); Modified Fatigue Impact Scale (MFIS)^75^ for perceived fatigue evaluation and the oral version of the Symbol Digit Modalities Test (SDMT) for cognitive functioning^76^.

Informed consent was obtained according to a procedure approved by the local ethic committee (Comitato Etico Regionale Liguria, IRCCS Azienda Ospedaliera Universitaria San Martino—IST, Genoa, Italy; P.R. 258REG2015) and to the Declaration of Helsinki.

### Apparatus

A digitizing tablet, the SMART TAB (E.M.S., S.r.l., Bologna), was used to acquire handwriting movements. The SMART TAB consists of a touch-sensitive tablet, a USB controller box, and a cable that connects the tablet with the controller. The controller box is connected via USB cable to the PC that manages the acquisition. The software handling the acquisition of the writing movements was developed in our laboratory in MatLab® platform (Psychophysics Toolbox)^77,78^. It provides a visual feedback of what the subject is writing on a computer monitor while recording the written trace. Details on hardware and software used during this experiment can be found in a previous study of our group^79^.

### Experimental procedure

Participants were seated on a chair at a table. The SMART TAB was placed on the table and the participant could freely adjust its position to feel as comfortable as possible during writing that was performed with the right hand. Once a comfortable position was achieved the participant was requested to keep the orientation of the tablet and of the forearm constant. In order to mimic a conventional handwriting condition while recording movement kinematics, a sheet of paper with black horizontal lines was positioned over the SMART TAB surface and the subject was provided with a real ink pen. Thus, the participant had a visual feedback of what she/he was writing over the sheet of paper and could appreciate the normal friction of pen in the act of writing; in the meantime, the tablet acquired the trajectory of the pen and the experimenter could simultaneously monitor the writing motor output on the computer screen. The subject was asked to write three times, on three different lines, at spontaneous speed, the Italian sentence “Il sole scalda” (i.e., “The sun warms”) when a “go” signal was provided by the experimenter. This sentence was chosen because it was composed of simple words very common in Italian language.

### Data analysis

#### Data treatment

The kinematic parameters of handwriting movements were computed by means of a custom-made MatLab® software. The handwriting performance was assessed considering the sentence as a whole, but also the words and the spacing between words (i.e., the distance between one word and the following one). The software automatically detected the beginning and the end of the sentence on the basis of the module of the velocity profile (computed over the two dimensions of the tablet, x and y): the first and the last instants (in each line) in which the velocity was greater than zero value corresponded to the beginning and the end of the sentence, respectively. The software was used also to segment each sentence in words and spacing between words.

In order to provide a spatiotemporal description of the subjects’ performance we considered the following outcome parameters: the duration (s), the length (mm) and the height (mm) of the sentence, the duration and the length of the words, the duration and the length of the spacing between words. The duration of the sentence corresponded to the time employed by the subject to write an entire sentence; the length of the sentence was calculated as the size of the segment which connects the first point of the first word to the last point of the last word; the height was computed as the vertical distance between the top of the highest letter and the bottom of the lowest one. The duration and the length of the words were computed as the sum of the time spent to write the three words and the sum of the spatial distances covered by the three words, respectively, excluding the spacing between words. The sum of the time intervals elapsed between the end of the first and the beginning of the second word, and between the end of the second and the beginning of the third word is the spacing between words duration (s). Similarly, the sum of the length of the spaces between one word and the following one represents the spacing between words length (mm). In order to evaluate subjects’ variability we computed for each of the previously mentioned parameter the coefficient of variation (CV) as the ratio of the standard deviation to the mean.

The quality and the level of automation of handwriting was evaluated by means of the single stroke analysis^20,21^. A stroke is defined by the time segment between two subsequent changes in the vertical direction. Zero-crossings in the sentence velocity profiles, automatically identified by means of a custom-made MatLab® software, were used to define the limits of each up- or down stroke. Only strokes exceeding a duration of 50 ms and an amplitude of 1 mm were included in the analysis^36^. The software computed the following parameters: number of stroke (NoS), stroke duration (ms), stroke size (mm), normalized jerk (computed according to^52^; the low the jerk value the high the movement smoothness), number of inversions in the velocity profile (NIV; in skilled writers a single stroke is associated to NIV = 1^80^, NIV > 1 means that a stroke is produced by several sub-movements). As for the sentence parameters, CV was computed for the previously mentioned variables.

#### Statistical analysis

We checked that variables were normally distributed (Shapiro-Wilk test) and that sphericity was respected (Mauchly’s test). All the kinematic variables resulted normally distributed as also SDMT, MFIS, grip strength, Tri-Pod pinch strength, NHPT. The three VAS scores and Abilhand score were not normally distributed. A series of multivariate analysis of variance (MANOVAs) were conducted to compare the kinematic variables of the sentence and of the strokes between PwMS and HC groups to avoid a Type I error. In particular, a MANOVA was conducted with mean sentence duration, length and height, mean duration and length of the words and of the spacing between words, as dependent variables, and group as between-subject factor. The same analysis was repeated on the CV of the previously mention dependent variables. Then, a MANOVA was performed on stroke parameters; NoS, stroke duration, stroke size, normalized jerk, and NIV were the dependent variables, whilst group was the between-subject factor. Again, the same analysis was repeated on the CV of the stroke variables. Furthermore, for each MANOVA, we performed a post-hoc analysis (an ANOVA for each parameter) to find out which variables were significantly different between HC and MS. Bonferroni’s correction for multiple comparisons was applied. In the case of the sentence parameters we considered p-values < 0.05/7 = 0.007 statistically significant. In the case of the stroke parameters we considered p-values < 0.05/5 = 0.01 statistically significant.

Kinematic data of PwMS group that resulted to be significantly different from those of the control group were correlated with the scores obtained during the clinical evaluation. Multiple linear regression (MLR) models or Spearman’s correlation analysis were applied depending on data distribution. Collinearity was assessed for MLR model. The commonly utilized variance inflation factor (VIF) and Tolerance techniques were adopted to detect predictor variables that were highly inter-correlated, creating redundancy in predictors and subsequent proportion of variance accounted for. Variables were dropped if the VIF was greater or equal to 10 or the Tolerance was less than 0.1.

### Data Availability

Please contact the corresponding author for data requests.
